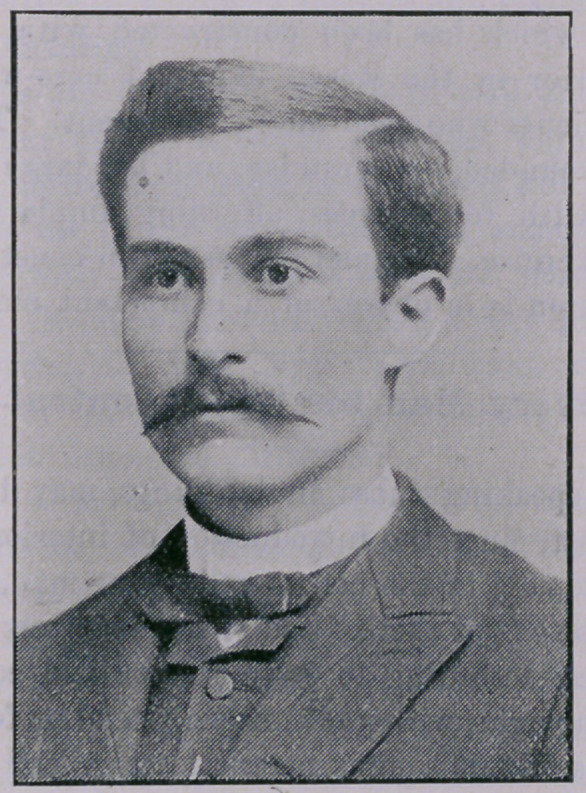# Medical News and Miscellany

**Published:** 1899-04

**Authors:** 


### Medical News and Miscellany.

**Dr. W. E. Davis** has removed from Elkhart to Guy's Store, Texas.

---

**Dr. A. C. Mecom** has removed from Perryman, Texas, to Tarkington's Prairie, Liberty County, Texas.

---

**Dr. I. J. Jones,** of Austin, late surgeon to the Texas Confederate Home, has been appointed Secretary of the Quarantine Department.

**T. B. Reams, Esq.**—This is a good picture of our friend, T. B. Reams, the Texas representative of the old and well known drug and chemical house of Wm. R. Warner & Co., of Philadelphia, whose specialties are so popular everywhere. Mr. Reams is a native of Tennessee, has a good literary education, and has studied but not practiced—medicine. He is one of the best posted men in pharmacy and therapeutics now on the road. He is a thorough-going drug man; in fact, in the vernacular, a rustler. Moreover, he is genial, of an attractive social turn, even tempered,—always in a good humor; hence his popularity with doctors and druggists. When Reams calls on you, doctor, give him the "cordial"—he's the right sort.

---

**The Medico-Chi. Wins.**—The Medico-Chirurgical College petitioned Common Pleas Court No. 3 for leave to amend its charter so as to grant the diplomas and degrees in dental surgery, etc. This was resisted by the Philadelphia Dental College on the ground of want of authority to do so, etc. The Common Pleas Court decided in favor of the Medico-Chi., and the dental college took an appeal from his decision. The Supreme Conrt, in an opinion by Judge Dean, has just confirmed the decision of the lower court, and dismissed the appeal.

**Drs. Jelks and Holland,** editors of the *Hot Springs Medical Journal*, opened the Ozark Sanatorium, Hot Springs, Ark., March 15th, for the reception of patients. The building is four stories high, with hydraulic elevator. It has bath tubs on each floor, and in addition a large bath room in the basement. It has a government hot water privilege, and the sanitary and hygienic arrangements of the building are perfect. It has a well appointed surgical department, fitted up with the latest appliances, the operating room of which has been constructed with great care, and has not a superior in the west. Especial care will be taken to provide for patients who need dietic treatment. The Sanatorium building is surrounded by verandas, and has large grounds, beautifully shaded with forest trees, affording ample opportunity for fresh air and exercise. Trained nurses are in constant attendance, and the institution is in charge of a competent matron.

---

### Every Man his own "Central."

The use of speaking tubes in buildings may be considered a thing of the past, since the introduction of interior telephone systems. A simple ring of the bell to call "your man," who "answers back" to show he's "got there," and you are conversing in dulcet or stentorian tones, according to your temper and courage—face to face, bold and upright, with your unseen friend or foe. No Tommy Piper's act as a "25,000 ohms magneto tester" of your lungs nor danger to your blood vessels, nor a stiff neck, in the attempt to practice on the small boy's delight. And you need not stir an inch, just sit firm, place the handy little desk set at your elbow, adjust the switch, and by a simple little twist of your wrist eject an electric current from your generator into the ringer coils, and the man you want "gives it to you" back, without a shock. You have "got him on" the wire, although he may be a little sassy at a distance. "Ring off," hang up the receiver, and the deed is done.

The idea is suggested by the advertisement of Mr. Proal Judson, State Sales Agent at Galveston of the Viaduct Manufacturing Company of Baltimore, large manufacturers of telephone outfits, fire alarm, district telegraph and general electric apparatus.

About two summers ago the Viaduct Company installed and presented, through Mr. Judson, two of their interior intercommunicating telephone systems to the Young Men's Christian Association and the Garten Verein in Galveston. One system is operated through a seven drop switchboard—a miniature exchange outfit—

while in the other each station has its own central office and has automatic switch attachment whereby the act of replacing the receiver forces the switch to its normal position, thus preventing any station from being left out of circuit. The latter system is also now made with the switch so arranged that no matter where it may be left calls will come in, and it is only necessary to see that the switch is placed upon the home stud in order to talk. This system is simple and positive of action, while the automatic switch requires rather careful handling.

These systems are furnished with both wall and desk sets, and have proven their utility in stores, factories, warehouses, colleges, hospitals, etc., where more than two stations are required. They are in extensive use in the large cities, and among the many concerns equipped with them in Baltimore, the most notable is the *Baltimore Sun*. Even on the Pacific coast a very large number have been placed during the past three years, some with as many as thirty stations, by the company's representative, Paul Seiler Electrical Works, San Francisco, California.

For private houses they have perfected an inexpensive, handy little instrument for communication from the different rooms to the kitchen with annunciator service.

Messrs. J. Moller & Company, a prominent shipping firm in Galveston, have seven of the company's multiple bell telephones on a metallic circuit connecting their private and general offices with an extensive telephone and signal bell system on their wharf where their ships load. These bells are made especially for long distance to ring through many stations on the same line, and are used largely on cross country or territorial lines.

The works of the Viaduct Company are about nine miles out of Baltimore, at Relay Station on the Baltimore & Ohio Railroad, situated near a high arch stone viaduct, from which the company takes its name, and over which the railroad crosses the river supplying the power to run the machinery.

The capacity of the works for making telephones is about two hundred per day, and the output of district messenger call boxes is eight thousand to ten thousand a year, besides fire alarm systems, trolley signal outfits, annunciators, etc. Over sixty thousand call boxes have been made for the Postal Telegraph Co. The number of hands employed is about one hundred and twenty-five on an average. The works include twelve buildings, some being residences for the employes, surrounded by a tract of twenty-five acres owned by the company.

Mr. A. G. Davis, the president, has had long experience in electric lines, having learned telegraphy in 1849 under the instruction of his cousin, Samuel F. B. Morse, the pioneer in message transmission, and has charge of a number of telegraph lines, including the original equipment of the Grand Trunk Railway in Canada.

Samples of some of the company's output can be seen at Mr. Judson's office, at Galveston, in the Levy Building, corner Tremont and Market Streets, and orders can be sent through him direct, or any of the local electrical dealers.

### Special Noiice.

DOCTOR.—Read the book reviews. We give this month a criticism on a good many new and important works, just from the press. They are for sale, and as there is only one copy of each, if you see any book mentioned in this department at any time that you need, order it at once. Cash with order, 20 off publisher's price. They are all cloth.

## Figures and Tables

**Figure f1:**